# Silica Nanoparticles Effects on Blood Coagulation Proteins and Platelets

**DOI:** 10.1155/2016/2959414

**Published:** 2016-01-06

**Authors:** Volodymyr Gryshchuk, Natalya Galagan

**Affiliations:** ^1^ESC “Institute of Biology”, Kyiv National Taras Shevchenko University, 64/13 Volodymyrska Street, Kyiv 01601, Ukraine; ^2^Chuiko Institute of Surface Chemistry, National Academy of Sciences of Ukraine, 17 General Naumov Street, Kyiv 03164, Ukraine

## Abstract

Interaction of nanoparticles with the blood coagulation is important prior to their using as the drug carriers or therapeutic agents. The aim of present work was studying of the primary effects of silica nanoparticles (SiNPs) on haemostasis* in vitro*. We studied the effect of SiNPs on blood coagulation directly estimating the activation of prothrombin and factor X and to verify any possible effect of SiNPs on human platelets. It was shown that SiNPs shortened coagulation time in APTT and PT tests and increased the activation of factor X induced by RVV possibly due to the sorption of intrinsic pathway factors on their surface. SiNPs inhibited the aggregation of platelet rich plasma induced by ADP but in the same time partially activated platelets as it was shown using flow cytometry. The possibility of SiNPs usage in nanomedicine is strongly dependant on their final concentration in bloodstream and the size of the particles that are used. However SiNPs are extremely promising as the haemostatic agents for preventing the blood loss after damage.

## 1. Introduction

Interactions of nanoparticles with the blood coagulation system can be beneficial or adverse depending on the intended use of a nanomaterial. Nanoparticles can be engineered to be procoagulant or anticoagulant or to carry drugs to intervene in other pathological conditions in which coagulation is a concern [1]. Using of nanoparticles as the drug carriers is extremely promising and is of interest for scientists all over the world [2, 3]. Nanoparticles can be designed as bacteriostatic agents as well [4]. Silica nanoparticles (SiNPs) have been shown as a promising alternative for biomedical applications [5] as a novel delivery in tumor research vector or tumor-targeting agent [6, 7], and as the carriers of anticancer drugs [8, 9]. For the use of SiNPs in bioengineering the study of their biocompatibility including cell toxicity [10], immunotoxicity [11], and genotoxic effects [12] is essential. The aim of present study was the estimation of the primary effects of SiNPs on haemostasis* in vitro*.

## 2. Materials and Methods

### 2.1. Materials

#### 2.1.1. Chemicals

Chromogenic substrates S2238 (H-D-Phe-Pip-Arg-pNA) and S2236 (p-Glu-Pro-Arg-pNa) were purchased from BIOPHEN, and APTT-reagent was from Renam (Russia). Ecamulin purified from *Echis multisquamatis* venom was kindly donated by Dr. Korolova. Factor X activator from Russell's viper venom (RVV) and ADP were purchased from Sigma (US).

#### 2.1.2. Silica Nanoparticles

Amorphous silica nanoparticles (SiNPs) A300 (Kalush, Ukraine) were pretreated at 400°C during 2 hours. The resulting material had the average space of surface as approximately 300 m^2^/g and the size of particles ranged from 10 to 40 nm [13].

### 2.2. Methods

#### 2.2.1. Platelet Rich Plasma (PRP) and Blood Plasma Preparation

Venous blood of healthy volunteers who had not taken any medication for 7 days was collected into 38 g/liter sodium citrate (9 parts blood to 1 part sodium citrate). PRP was isolated by centrifugation of blood at 160 g for 20 min at 23°C. For the preparation of platelet poor blood plasma PRP was spinned-down at 1500 rpm during 30 min [14].

#### 2.2.2. Activated Partial Thromboplastin Time

Activated partial thromboplastin time (APTT) was performed according to the following procedure. 0,1 mL of blood plasma was mixed with equal volume of APTT-reagent and incubated during 3 minutes at 37°C. Then the coagulation was initiated by adding of 0,1 mL of 0.025 M solution of CaCl_2_. Clotting time was monitored by the Coagulometer Solar CGL-2410 (Belorussia) [15].

#### 2.2.3. Amidase Activity Assay

Hydrolysis of chromogenic substrates (S2238, H-D-Phe-Pip-Arg-pNA, S2765, Z-D-Arg-Gly-Arg-pNA) under the influence of silica nanoparticles (SiNPs) was studied using the reader (Thermo-Scientific), E405–E492. The analysis was done in 0.05 M Tris-HCl buffer of pH 7.4 solution, 37°С. Chromogenic substrates were taken in the final concentration 30 mM. The final concentration of SiNPs was 0.4 mg/mL [16].

#### 2.2.4. Fibrinogen Concentration Study

Fibrinogen concentration in the blood plasma was determined by the modified spectrophotometric method. Blood plasma (0.2 mL) and PBS (1,7 mL) were mixed in glass tube. Coagulation was initiated by the addition of 0.1 mL of thrombin-like enzyme from the venom of* Agkistrodon halys halys* (1 NIH/mL) that allowed avoiding fibrin cross-linking [17]. Mixture was incubated during 30 min at 37°C. The fibrin clot was removed and resolved in 5 mL of 1,5% acetic acid. The concentration of protein was measured using spectrophotometer SF-2000 (Russia) at 280 nm (*ε* = 1,5).

#### 2.2.5. Platelet Aggregation Study

Platelet aggregation measuring was based on changes in the turbidity of platelet rich human plasma [18]. In typical experiment 0.4 mL platelet rich plasma was incubated with 0.02 mL of 0.025 M CaCl_2_ and ADP in final concentration 12.5 *μ*M at 37°C. Studied concentrations of SiNPs were 0,001 and 0,01 mg/mL. Aggregation was detected for 10 min using Aggregometer Solar AP2110.

#### 2.2.6. Flow Cytometry

The shape and granulation of platelets after incubation with silica nanoparticles (SiNPs) versus platelets activated by thrombin were monitored on COULTER EPICS XL Flow Cytometer [19]. SiNPs (0,01 mg/mL) were added to 1 mL of washed platelets suspension and samples were incubated during 90 min at 25°С. Scattered and transmitted light was monitored for examining any changes of the platelets granulation and shape.

#### 2.2.7. Statistic Data Analysis

Statistical data analysis was performed using Microsoft Excel. All assays were performed in series of three replicates and the data were fitted with standard errors using “Statistica 7.”

## 3. Results

### 3.1. Proteins of Coagulation System with SiNPs

Amorphous silica nanoparticles (SiNPs) were precipitate in the PBS with the final concentration of stock solution 2 mg/mL. All samples of SiNPs were mixed using Vortex* ex temporo*.

**Figure 1:**
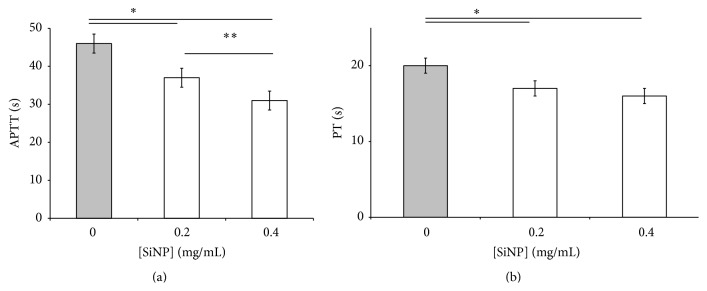
Activated partial thromboplastin time (APTT) of human blood plasma (a) and prothrombin time (PT) of human blood plasma (b) in the presence of 0,2 mg/mL and 0,4 mg/mL of SiNPs.

The basic coagulation tests APTT and PT were performed in the presence of SiNPs suspension in the final concentrations 0.2 and 0.4 mg/mL. It was shown that SiNPs distinctly shortened coagulation time in both tests in concentration dependant manner (Figure 1). The effect was more evident for PT than for APTT.

Specific chromogenic substrates to prothrombin (S2238) and factor Xa (S2765) have been used to avoid the influence of fibrinogen sorption by SiNPs on the results of the tests.

**Figure 2:**
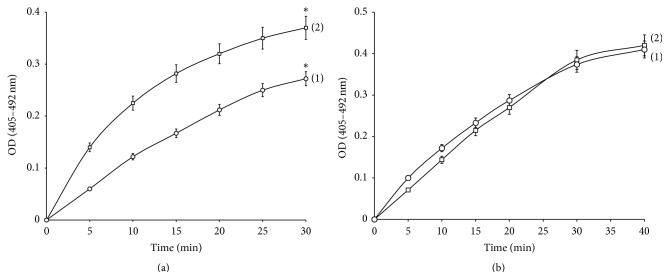
(a) Effect of SiNPs (0,4 mg/mL) on factor X activation by RVV. (b) Effect of SiNPs (0,4 mg/mL) on prothrombin activation by ecamulin. (1) Control; (2) sample with SiNPs.

We studied the effect of SiNPs on blood coagulation directly estimating the activation of prothrombin and factor X. Prothrombin was activated by the prothrombin activator from *Echis multisquamatis* venom (ecamulin). The resulting thrombin activity was detected by thrombin-specific chromogenic substrate S2238. It was shown that SiNPs taken in the concentration 0.4 mg/mL did not affect the direct activation of prothrombin (Figure 2(a)).

Factor X was activated directly by RVV [20], activity of activated factor Xa estimated with specific chromogenic substrate S2765. It was shown that 0.4 mg/mL of SiNPs inhibited the factor X activation (Figure 2(b)).

Thus we observed the inhibitory effect of SiNPs on blood coagulation that consisted in prolongation of time of coagulation initiated by thromboplastin and APTT-reagent as well as in the decreasing of the activation of factor Xa but not prothrombin.

### 3.2. Platelet Aggregation and Activation

**Figure 3:**
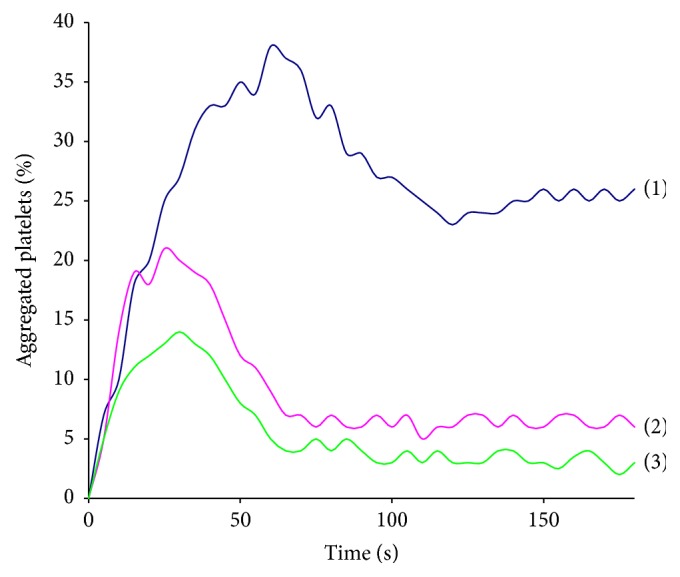
Platelet aggregation in platelet rich plasma (PRP) induced by 2.5 *μ*M ADP in the presence of 0.001 mg/mL and 0.01 mg/mL of SiNPs. (1) Aggregation of control PRP; (2) aggregation of PRP in the presence of 0.001 mg/mL of SiNPs; (3) aggregation of PRP in the presence of 0.01 mg/mL of SiNPs.

Platelet aggregation in PRP induced by ADP was studied in the presence of absence of SiNPs at the final concentrations 0.001 and 0.01 mg/mL. It was shown that these concentrations of SiNPs inhibited the rate and the speed of platelet aggregation (Figure 3). 0.001 mg/mL of SiNPs decreased the aggregation rate twice and provoked huge disaggregation of platelets. These effects were even more evident when 0.01 mg/mL of SiNPs was taken.

It is known that fibrinogen takes part in platelet aggregation by attracting the platelets to each other and to the newly formed clot. That is why we examined the ability of SiNPs to adsorb fibrinogen. It was shown that 1 mg of SiNPs adsorbed 0.325 mg of fibrinogen of blood plasma. Thus we can assume that the sorption of fibrinogen by SiNPs taken in the concentrations 0.001 and 0.01 mg/mL could not inhibit platelet aggregation by removing the plasma fibrinogen.

The inhibitory effect on platelet aggregation could be explained by the action of studied agent on intracellular signaling or/and on binding of fibrinogen with its platelet receptor IIbIIIa [21]. So we have to examine whether the SiNPs could inhibit the platelet activation. For this aim the activation of platelets induced by thrombin was monitored using flow cytometry. The platelet shape and granularity were studied by direct and orthogonal light scattering [18].

**Figure 4:**
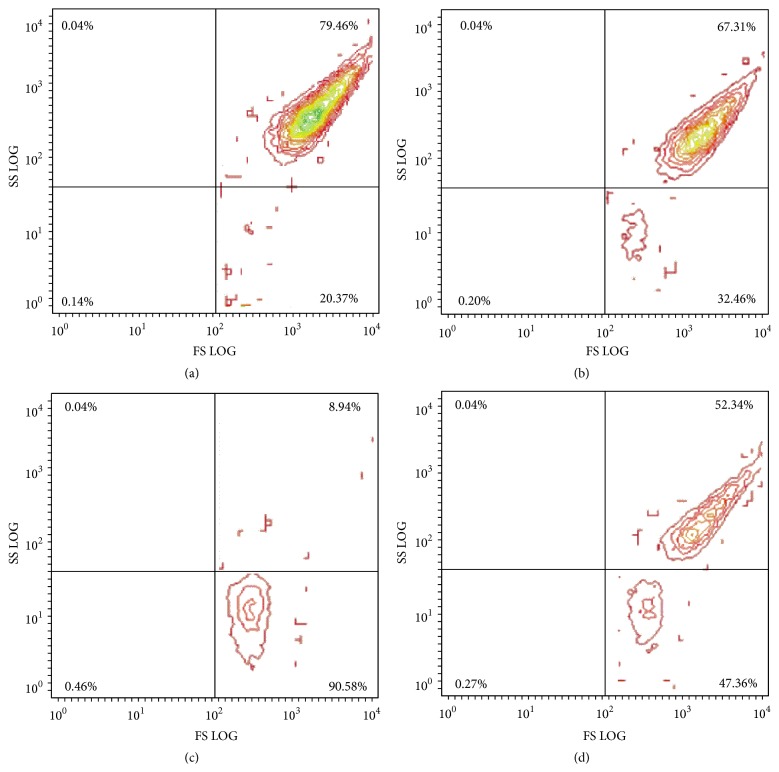
Flow cytometry of human platelet rich plasma incubated with 0.01 mg/mL and 0.001 mg/mL of silica nanoparticles (SiNPs). Distribution of platelets according to their shape and granulation. SS LOG, parameter of platelets granulation: FS LOG, parameter of platelets shape. (a) Resting platelets incubated with equal volume of TBS as a control; (b) resting platelets incubated with 0.001 mg/mL of SiNPs (silica nanoparticles) during 5 min; (c) platelets activated by thrombin (0,125 NIH/mL) for 2 min; (d) platelets activated by thrombin, preincubated with 0.01 mg/mL of SiNPs during 5 min.

To verify any possible effect of SiNPs on human platelets, PRP was analyzed by flow cytometry. Resting human platelets (a) were incubated with 0.001 mg/mL of SiNPs (b) or 0.01 mg/mL of SiNPs (d) during 5 min. It was shown that the population of resting platelets decreased from 80 ± 3% in control sample (a) to 67 ± 4% and 52 ± 2% in the samples with 0.001 mg/mL of SiNPs (b) or 0.01 mg/mL of SiNPs (d), respectively (Figure 4). In contrast, activation of platelets by thrombin (c) caused dramatic change to the platelets shape and granularity. Therefore we concluded that SiNPs were able to induce slight changes of shape and granularity of platelets that did not cause platelet aggregation but could affect platelet aggregation induced by ADP.

## 4. Discussion

Interaction of silica with blood coagulation proteins is well studied area. Previously it was shown that the silica activity depends on the particle diameter and consists in the geometrical relations between the silica and the protein molecules [22]. The conformation of adsorbed proteins on the colloidal silica surfaces plays a role in modulating the amount of their function and cell binding [23]. It was shown that SiNPs effectively adsorbed fibronectin, fibrinogen, and so forth. Therefore SiNPs also modulate processes of cell adhesion by absorbing adhesion molecules [24] and also by direct incorporation in blood cells [25]. It was reported that SiNPs penetrated the platelet plasma membrane and stimulated a rapid and prolonged NO release, IIbIIIa activation, and finally the platelet aggregation [25].

In our study we showed the procoagulant effects of silica reported in several studies and used in combat gauze development [26, 27] caused mainly by the action of SiNPs on factor Xa activation. Our previous findings reported in [28] showed that this activation did not occur when intrinsic coagulation factors (namely, XII and XI) were removed from the incubation volume. This data corresponds to the results of that showeing the sorption of factor XII on the surface of SiNPs that strongly depends on the size of the particles [29, 30]. However, any of our experiments showed the direct coagulant action of SiNPs on unactivated blood plasma or the activating action of SiNPs on platelet aggregation. Despite this, studied SiNPs were able to change the shape and granularity of resting platelets studied by flow cytometry that corresponded to the data of other scientists that demonstrated incorporation of SiNPs in the living cells [25]. We can assume that small SiNPs from the studied samples (10 nm) were incorporated into cells and activate them (as it was shown by Corbalan et al. [25]) but bigger nanoparticles (up to 40 nm) that also were present in the samples inhibited platelet aggregation by absorbing fibrinogen [23, 24] that is sufficient for platelets aggregation [21].

Our findings also corresponded to the results obtained recently on* in vivo* models of SiNPs administration where systemic activation of coagulation cascade and platelets were shown [30, 31].

## 5. Conclusions

Amorphous SiNPs are able to increase the activation of coagulation cascade by adsorbing and stimulating of intrinsic pathway coagulation factors. This effect resulted in shortening of coagulation time in APTT and PT tests, as well as in the increasing of factor X activation by RVV in blood plasma but not in the sample with removed factors XI and XII. SiNPs did not induce platelet aggregation in PRP but changed the shape and granularity of resting platelets and inhibited their aggregation. The possibility of SiNPs usage in nanomedicine is strongly dependant on their final concentration in bloodstream and the size of the particles that are used. However, SiNPs are extremely promising as the haemostatic agents for preventing the blood loss after damage.

## Abbreviations

SiNPs: Silica nanoparticles
RVV: * Russel vipera* venom
PRP: Platelet rich plasma
APTT: Activated partial thromboplastin time
PT: Prothrombin time.
